# New insights into the origin and evolution of α-amylase genes in green plants

**DOI:** 10.1038/s41598-019-41420-w

**Published:** 2019-03-20

**Authors:** Liangliang Ju, Zhifen Pan, Haili Zhang, Qiao Li, Junjun Liang, Guangbing Deng, Maoqun Yu, Hai Long

**Affiliations:** 1 Chengdu Institute of Biology, Chinese Academy of Sciences, Chengdu, 610041 China; 2 University of Chinese Academy of Sciences, Beijing, 100049 China

## Abstract

Gene duplication is a source of genetic materials and evolutionary changes, and has been associated with gene family expansion. Functional divergence of duplicated genes is strongly directed by natural selections such as organism diversification and novel feature acquisition. We show that, plant α-amylase gene family (*AMY*) is comprised of six subfamilies (*AMY1*-*AMY6*) that fell into two ancient phylogenetic lineages (*AMY3* and *AMY4*). Both *AMY1* and *AMY2* are grass-specific and share a single-copy ancestor, which is derived from grass *AMY3* genes that have undergone massive tandem and whole-genome duplications during evolution. Ancestral features of *AMY4* and *AMY5*/*AMY6* genes have been retained among four green algal sequences (Chrein_08.g362450, Vocart_0021s0194, Dusali_0430s00012 and Monegl_16464), suggesting a gene duplication event following Chlorophyceae diversification. The observed horizontal gene transfers between plant and bacterial *AMY*s, and chromosomal locations of *AMY3* and *AMY4* genes in the most ancestral green body (*C*. *reinhardtii*), provide evidences for the monophyletic origin of plant *AMY*s. Despite subfamily-specific sequence divergence driven by natural selections, the active site and SBS1 are well-conserved across different AMY isoforms. The differentiated electrostatic potentials and hydrogen bands-forming residue polymorphisms, further imply variable digestive abilities for a broad substrates in particular tissues or subcellular localizations.

## Introduction

As the best known and most deeply studied amylolytic enzyme^1–6^, α-amylase (AMY, α-1,4-glucan-4-glucanohydrolases, EC 3.2.1.1) is an ubiquitous hydrolase synthesized by plants, animals and microorganisms, catalyzing the cleavage of internal α-(1–4)-glycosidic linkages in starch, glycogen and other related oligosaccharides with a retaining endo-acting mechanism. Its widespread distribution reflects the use of principal energy and carbon source through exploiting environmental polysaccharides. Currently, under the sequence-based classification rules of carbohydrate-active enzymes (CAZy database, http://www.cazy.org/index.html), AMYs are classified as the main representative of the glycoside hydrolase (GH) family 13, and probably also present in families GH57, GH119 and GH126^5–7^. These enzymes in GH13 are characterized by adopting the (β/α)_8_-barrel (TIM-barrel) catalytic domain, and display strong conservation of their tertiary conformation, although the amino acid sequences exhibit a high degree of variability, and only a few amino acids are conserved, as revealed by inter-kingdom pairwise comparisons^1–3,5,6,8,9^; however, the sequence similarities are much higher within kingdoms. Our present work focuses on the AMYs belonging to GH13.

The evolution of *AMY* genes has been an attractive subject for more than 30 years. The original study was presented by Nakajima^1^, who compared 11 different *AMY* sequences from plants, animals and microbes, and observed four highly conserved regions necessary for enzyme functions. Subsequently, Janecek^2^ analyzed 37 sequences that were also from the different living organisms, and established three main phylogenetic lineages: fungi and yeasts, plants, and streptomycetes, *Thermomonospora cuwata*, insects and mammals. The archaea *AMYs* showed close relatedness with their plant counterparts, and these two distinct branches should retain their own originality^10^. Additionally, several bacterial *AMYs* have been reported to share the typical animal-like motifs and chloride-dependent properties^2,9,11–15^. This observation raised the α-amylase model of horizontal gene transfer (HGT) between animals and bacteria^9^. However, the origin of animal *AMY* genes is still debatable under alternative hypotheses^4,16^. Within the family GH13, *AMYs* from different living groups were separately present in particular subfamilies, such as GH13_1 (fungi and yeasts), GH13_6 (plants), GH13_7 (archaeons), GH13_24 (mammals), etc (CAZy^5^). It was worthy to note that the bacterial *AMYs* were rather scattered in several clusters, such as the plant/archaea-type bacterial *AMYs*, the animal-type bacterial *AMYs*, the fungi/yeast-type *AMYs*, and the bacterial-type *AMYs* without any strict similarity with the other living organisms^3,9^.

In humans and other mammals, there are two divergent paralogous loci (*AMY1* and *AMY2*) responsible for the tissue-specific production of salivary and pancreas α-amylases, respectively^17–21^. From the perspective of evolution, these two loci should be generated through tandem duplication from one ancestral copy^17^. One of them, the salivary *AMY1*, was selected by evident adaptive pressures, such as the behavioral variation of starch consumption^22,23^. Among *Drosophila* species, the *AMY* gene family of *D*. *ananassae* is believed to be the most complicated situation in animals, because its seven copies are distributed on the different chromosomes, and mainly organized as two genetically distinct clusters; and two copies of them, *Amyrel* and *Amyc1*, shows strong sequence divergence with the two classical paralogous clusters^24,25^.

In green plants, extensive studies of *AMY* genes have been focused on the grass lineage (i.e., wheat, barley and rice), because these genes are of critical importance to seed germination and grain maturation. Based on the biochemical isoelectric point (pI), the high-pI (*AMY1*) and low-pI (*AMY2*) genes were systematically characterized in wheat and barley^26–29^. *AMY3* was initially identified in wheat but not in barley^30^, and recent transformation experiments indicated that it was expressed with the most abundance in developing wheat grains^31^. In rice, a total of ten genes have been classified as five hybridization groups, corresponding to three subfamilies: *RAmy1* (*A*, *B* and *C*), *RAmy2A*, and *RAmy3* (*A*, *B*, *C*, *D*, *E* and *F*)^32,33^. Subsequent phylogenetic inference indicated that cereal *AMY* genes fell into two classes: *AmyA* (*AMY1* and *AMY2*) and *AmyB* (*AMY3*), and they should be derived from a duplication event of the common ancestor of monocot and dicot *AMY* genes^34^. In bread wheat, Mieog *et al*.^35^ recently characterized four α-amylase genes (*TaAmy1*-*TaAmy4*). However, for a detailed phylogenetic of cereal *AMY* genes, additional evidence collected from a larger taxonomic scale is needed. Wegrzyn *et al*.^36^ isolated an apple *AMY* gene (*AMY8W*), which showed most similar to a gene (GenBank accession M79328) from potato. These two genes lacked the standard signal peptides, and formed a distantly separate branch, which appeared to have diverged prior to the split of monocot and dicot plants. In *Arabidopsis*, three *AMY* genes (*AMY1*, At4g25000; *AMY2*, At1g76130; and *AMY3*, At1g69830) have been well-annotated, and only At1g69830 is predicted to be leaf chloroplasts targeting^37^. Unlike the monocot *AMY1* to *AMY3* genes, the newly described barley *AMY4* showed nearly 70% sequence identities to several dicot *AMYs*, including those from *Plantago major*^38^, *Malus domestica*^36^ and *Arabidopsis AMY2*^39^. Furthermore, a recent clustering analysis subdivided it into two distinct subgroups (*AMY4-1* and *AMY4-2*) across grass species^40^. Structural studies indicated that the overall crystallography of cereal α-amylases (AMY1 and AMY2) consisted of three domains: a central conserved (β/α)_8_-barrel domain (domain A), an additional domain B nested between β_3_ and α_3_ of domain A, and a five-stranded C-terminal β-sheet domain (domain C)^41–43^. The active site and starch granule binding surface site (SBS1) are conserved in most reported enzymes; and the surface binding site of domain C (SBS2) is unique in low-pI AMY2^42^.

Multiple isoforms encoded by different *AMY* genes suggested that their roles in starch degradation might depend on specific plant tissues or subcellular types. In germinating cereal seeds, AMYs are typical secretory protein molecules, that biosynthesized in the secretory tissues (the scutellar epithelium and the aleurone layer), and secreted subsequently into the starchy endosperm^44^. Radchuk *et al*.^39^ detected AMY4 isoforms in tissues that undergo programmed cell death (PCD) during caryopsis development. In contrast with secretory isoforms, the *Arabidopsis* AMY3 has a predicted N-terminal transit peptide for chloroplasts localization, and is involved in leaf transitory starch degradation^45,46^. Additionally, it was reported to possess two carbohydrate-binding module 45 (CBM45) repeats^47^. In rice, the isoform α-amylase I-1 is a typical secretory glycoproteins encoded by *RAmy1A*^48^, and involved in starch degradation in leaf chloroplasts^49,50^. It has also been shown that elevated activation of α-amylase I-1 by high temperature cause the unacceptable grain chalkiness in developing grains^51^. Recently, Mitsui *et al*.^52^ presented an comprehensive review, providing new insights into functional roles in intracellular transport, organelle targeting, and organ-specific actions. In potato tubers, two *AMY* genes (*StAmy1* and *StAmy23*) were expressed, but only *StAmy23* could be induced by low temperature^53^. Recently, Hou *et al*.^54^ indicated that StAmy23 degraded the cytosolic phytoglycogen in sweetening tubers.

Plant starches are the principal storage reserve constituents, and their degradations need the coordinated participations of categories of amylolytic enzymes in distinct tissues (i.e., leaves, tuberous tissues and endosperms). The wide-distribution nature of α-amylases in the main living organisms suggests its central importance in basal carbohydrate metabolisms, such as the glycogen and starch degradation. Previous studies conducted extensive inter-kingdom comparisons of AMY enzymes/genes, and successfully extracted conserved sequence motifs responsible for enzyme specificity and/or function. However, available data is largely lacked within kingdoms, especially in green plants. The increasing megabase genome datasets enable us to conduct the first comprehensively analysis of *AMY* genes with sampling from green algae to higher angiosperms. We hope this work can contribute to a better understanding of the origin, expansion dynamics, and functional diversification of this multiple gene family.

## Results

### AMY genes in green plants

**Figure 1:**
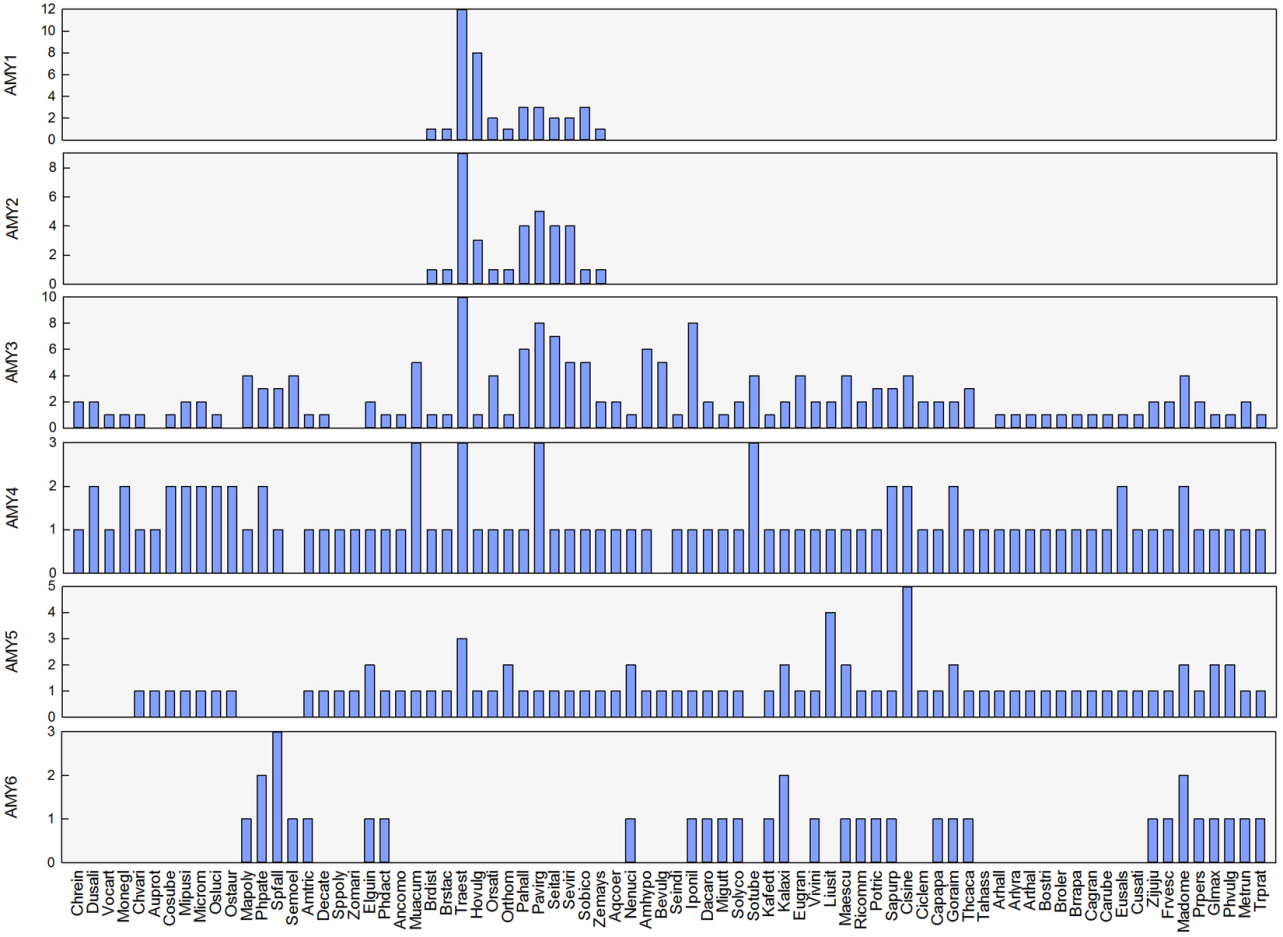
Distribution and copy number of *AMY* genes in green plants.

Our similarity searches resulted in 472 AMY or AMY-like sequences from the 78 investigated green species (Table S1). The global sequence alignment clearly divided the α-amylase gene family into six subfamilies: *AMY1* (CAX51374-type), *AMY2* (CAX51372-type), *AMY3* (AAA34259- and AT4G25000-type), *AMY4* (CAX51375- and AT1G76130-type), *AMY5* (AT1G69830-type) and a novel subfamily *AMY6* which has not been described before (Fig. 1). The overall amino acid sequence identities ranged approximately from 36% to 76% between matching conserved regions. These subfamilies were conserved to possess the basic catalytic and C-terminal beta-sheet domains, while their N-terminal ends exhibited larger sequence variations, and carried different categories of signal peptides or other domain modules (Fig. S2). Among them, *AMY3*, *AMY4* and *AMY5* genes were widely distributed across the entire green lineage, while both *AMY1* and *AMY2* were grass-specific. *AMY6* genes were scattered around species of basal land plants (*Marchantia polymorpha*, *Physcomitrella patens*, *Sphagnum fallax* and *Selaginella moellendorffii*), basal angiosperms (*Amborella trichopoda*) and two monocots (*Elaeis guineensis* and *Phoenix dactylifera*), however, it was found in almost all the main dicot lineages, except the Citrus and Brassicaceae. Occasionally, the *AMY4* genes were absent in two species, *S*. *moellendorffii* and *Beta vulgaris*. We also had not detected any *AMY3* gene among the five species (green algae *Auxenochlorella protothecoides* and *Ostreococcus tauri*, the monocot *Spirodela polyrhiza* and *Zostera marina*, and the dicot *Tarenaya hassleriana*).

### Phylogenetic relationships of plant *AMYs*

**Figure 2:**
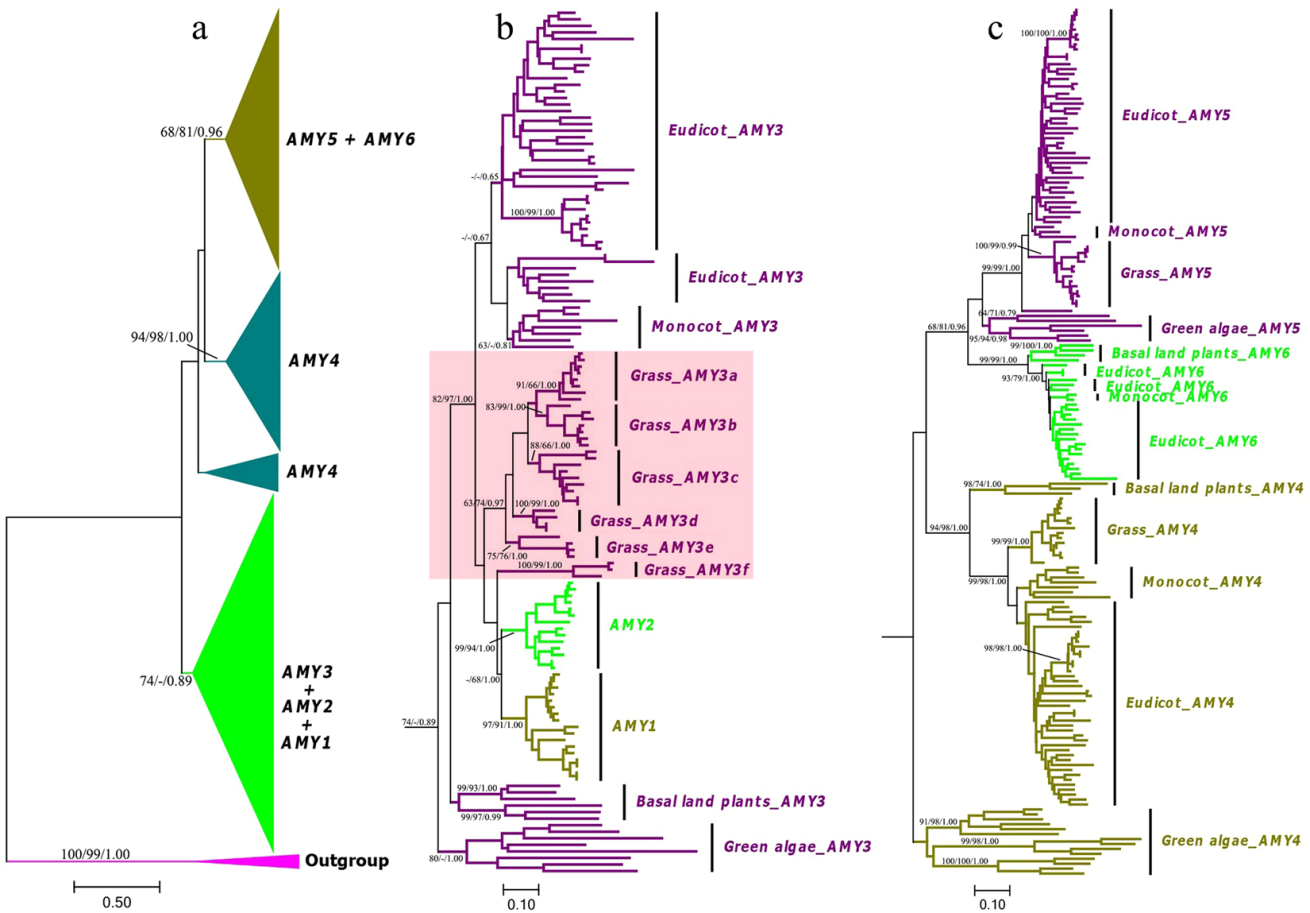
Phylogenetic relationships of *AMY* gene subfamilies in green plants. The phylogeny was inferred from combined analyses of NJ, ML and BI methods. Statistical supports associated with branches (>50%) were consecutively displayed as NJ and ML bootstrap values, and Bayesian posterior probabilities (BPP). The tree was rooted to bacterial *AMY* genes. (**a**) The overall NJ tree of *AMY* genes. (**b**) The subtree diagram of *AMY1* + *AMY2* + *AMY3* in (**a**). (**c**) The subtree diagram of *AMY4* + *AMY5* + *AMY6* in (**a**).

We used three different methods (NJ, ML and BI) to infer the phylogenetic relationships. The general topology placed plant *AMY* genes onto two major groups: *AMY1* + *AMY2* + *AMY3* and *AMY4* + *AMY5* + *AMY6* (Fig. 2a). In each subfamily, the phylogeny (i.e., *AMY3*, *AMY4*, *AMY5* and *AMY6*) exactly agreed to the green plant tree of life that is evolved from more ancient green algae or basal land and vascular plants, to higher angiosperms (Fig. 2b,c).

**Figure 3:**
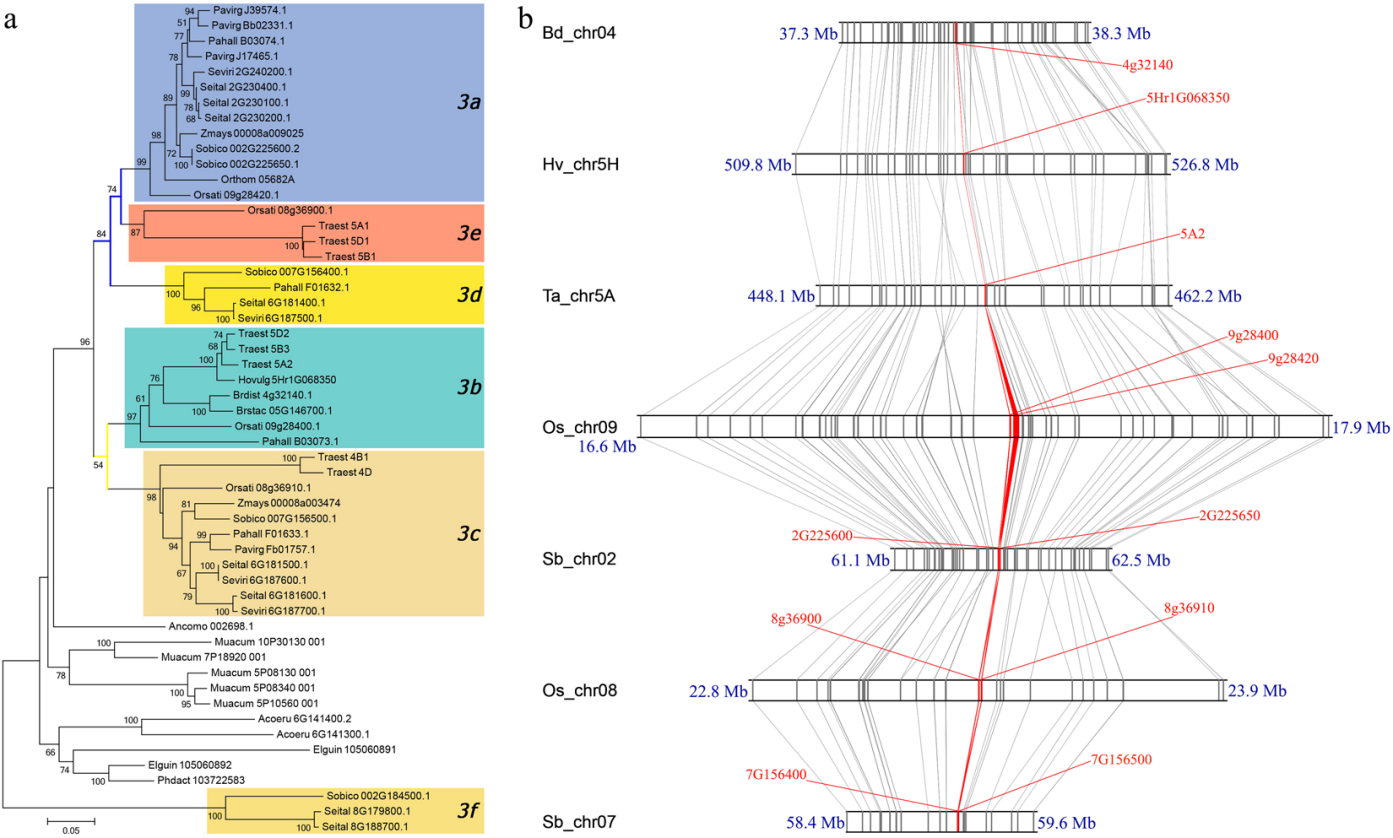
Phylogenetic and syntenic relationships of grass *AMY3* genes. (**a**) The NJ tree of *AMY3* genes, including all the *AMY3* copies from grass and other ancient monocot species. Branch supports (>50%) were displayed. (**b**) The conserved genic segments carrying the archetype *AMY3* loci that were highlighted with red color.

Within the subtree of *AMY1* + *AMY2* + *AMY3*, we defined six *Grass_AMY3* subclades (*3a* to *3f*) (Fig. 2b). To gain a more precise picture of these six subclades, we selected the redundant monocot and grass *AMY3* genes to re-evaluate their phylogeny and detected the synteny. We found subclades *3a*, *3e* and *3d*, and *3b* and *3c* were separately clustered together, and distinct from each other; the subcalde *3f* situated between *AMY3* and *AMY1* + *AMY2* (Figs 2b and 3a). All the single-copy genes (i.e., Hovulg_5Hr1G068350, Brdist_4g32140 and Traest_5A2) in diploid species belonged to the cluster of *3b* + *3c* (Fig. 3a).

Synteny detection indicated that *3b* + *3c* and *3a* + *3e* + *3d* were extremely conserved on chromosomes, and resulted from tandem gene duplications (Fig. 3b). Nevertheless, apparent gene colinearities were also observed on the chromosomes of Os_chr08 and Sb_chr07, which were duplicated from Os_chr09 and Sb_chr02 at the whole genome scale^55,56^. Subfamilies *AMY1* and *AMY2* were well segregated with each other, and formed a distinct branch that was embedded in the *AMY3* subtree (Fig. 2b). Our previous work demonstrated that the *AMY1* loci were conserved in a syntenic block, which were derived from the intermediate ancestral chromosome A2^57^. From this subtree, we found that *AMY1 and AMY2* located in an approximately equivalent position. Thus, we further detected the syntenic relationships of *AMY2* genes, and the genomic segments carrying the *AMY2* loci were also highly conserved and originated from A6 (Fig. S3).

There are three main clusters in the subtree of *AMY4* + *AMY5* + *AMY6* (Fig. 2a). Unexpectedly, the latter *AMY4* cluster (*Green algae_AMY4*) situated at a position that was distinct from *AMY4* genes of basal land plants to angiosperms, but with less powerful supports (Fig. 2c). Subfamilies *AMY5* and *AMY6* showed a closer relationship to each other than to *AMY4* (Fig. 2c). In the 11 sampled green algae species, the *Green algae_AMY5* genes were present in seven of them, while *AMY6* genes were absent in any of them (Fig. 1). By contrast, *AMY5* genes were absent in the four basal land species, but all of them contained the *AMY6* gene members (Fig. 1). Furthermore, these two subfamilies were featured by possessing the N-terminal extension of 460–570 amino acids, on which the chloroplast transit peptides were predicted (Fig. S2). Therefore, *AMY6* indeed represents a novel gene subfamily, duplicated and diverged from *AMY5*.

### Inter-kingdom sequence analysis

**Figure 4:**
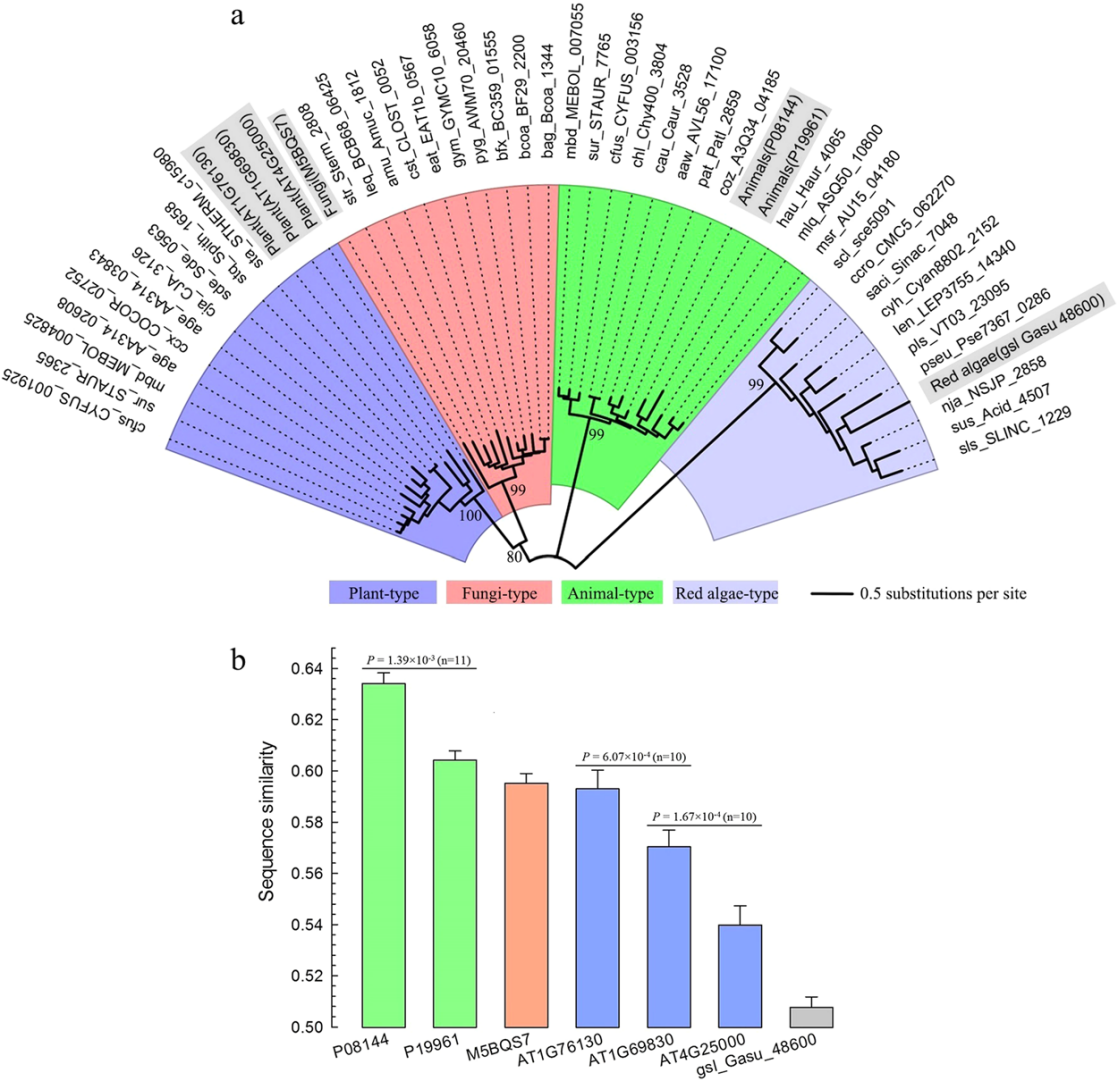
Analyses of *AMY* genes from different living organisms. (**a**) Close relationships of bacterial *AMY* genes with the other living groups. The representative query sequences were highlighted. The five main branch supports (>50%) were displayed. (**b**) Sequence similarities between queries and their bacterial counterparts. Significance values were calculated using paired two-sample t-test. Error bars represent SE.

Phylogenetic inference indicated that *AMY1* + *AMY2* + *AMY3*, *AMY4*, and *AMY5* + *AMY6* were three main *AMY* lineages in plants (Fig. 2). To further understand their origination, initially, we checked the *AMY* genes in the more ancient red algae, *Galdieria sulphuraria*. Interestingly, the amino acids of Gasu_48600 (Table S2) exhibited greater sequence divergence even than the bacterial outgroup when comparing with plant *AMY*s. Thus, we conducted sequence comparisons across different living organisms, and successfully extracted ten plant-type bacterial *AMY* genes. (Fig. 4a). In addition, the *AMY4* representative gene (At1g76130) showed the highest sequence similarity to plant-type bacterial *AMY* genes (Fig. 4b).

### Evolutionary pressures

Considering the wide distribution and neutral phylogenetic position of *AMY4*, we hypothesized that it might be the most basic subfamily. Thus, in the predefined tree, it was constantly treated as the background branch, and the other subfamilies were consecutively used as foreground branches; *AMY3* was defined to consist of *Eudicot_AMY3* and *Monocot_AMY3* (Fig. S1). Firstly, we estimated the whole tree using the one-ratio model (M0) and the nearly neutral site model (M1a). The estimates (ω_0_ = 0.10958, and ω_0_ = 0.10276 and p_0_ = 0.91084, respectively) indicated that *AMY* genes were deeply under selection constraints or purifying selections. In two-branch tests, all the LRT comparisons generated significant statistics with the exception of two-branch (*AMY6*), indicating the existence of varied ω-values across subfamilies (Table 1).

**Table 1:** Positive selection analysis using the Maximum likelihood method.

Models	*l*	LRT	Positive Selected Sites
M0: one-ratio	−26184.75	NA	NA
Two branches (*AMY1*)	−26174.68	7.20E-06	NA
Two branches (*AMY2*)	−26166.64	1.76E-09	NA
Two branches (*AMY3*)	−26179.54	1.25E-03	NA
Two branches (*AMY1* + *AMY2* + *AMY3*)	−26179.54	1.25E-03	NA
Two branches (*AMY5*)	−26174.38	5.26E-06	NA
Two branches (*AMY6*)	−26184.57	0.55	NA
Two branches (*AMY5* + *AMY6*)	−26181.63	1.43E-02	NA
M1a: nearly neutral	−26017.13	NA	NA
Model A (*AMY1*)	−25988.90	6.07E-13	p_2_ = 0.0481 (*P* > 0.52, 45P*/90T**/184S**/194N*/316K**)
Model A (*AMY2*)	−25975.19	0.00E + 00	p_2_ = 0.0799 (*P* > 0.54, 141P**/152N**/182N*/195D*/198Y*/248K**/251Q*/276R*/327G**/329T**/337A**/341L**)
Model A (*AMY3*)	−25993.51	6.09E-11	p_2_ = 0.0575 (*P* > 0.61, 22S**/80S**/263G*/376A*)
Model A (*AMY1* + *AMY2* + *AMY3*)	−25979.01	0.00E + 00	p_2_ = 0.2907 (*P* > 0.52, 33H*/52K*/101C*/115C*/139L*/140N*/142R**/213G*/263G*/276R**/316K*/328N**/359D*/361G**/366F*/)
Model A (*AMY5*)	−25987.02	9.36E-14	p_2_ = 0.1387 (*P* > 0.51, 13K**/29A*/72A*/76K*/93R*/124L**/152N*/308E*/309W*)
Model A (*AMY6*)	−25981.39	3.33E-16	p_2_ = 0.1593 (*P* > 0.58, 11W*/51G*/69L*/79K*/112S**/204D*/282S**/313S*/324N*/357K**/358Q**/365N*/369A*/376A*)
Model A (*AMY5* + *AMY6*)	−26004.65	3.79E-06	p_2_ = 0.16947 (*P* > 0.50)

BEB significance levels: *0.05, **0.01. *l*: log likelihoods. NA: not allowed. Amino acids referred to 1st sequence: Arlyra_7G28170.

In contrast to the branch or site models, we carried out the branch-site tests (Model A). These tests successfully detected proportions of sites with ω_2_-values greater than 1, and the LRT statistics were significant for all the M1a-Model A comparisons (Table 1). For instance, when *AMY1* was used as the foreground, there existed about a proportion of 4.81% sites with *P* > 0.52 under potential positive selections, and some residues (45P*/90T**/184S**/194N*/316K**) reached significant levels under the BEB inference. Note that some sites in the test of Model A (*AMY5* + *AMY6*), although not existing significant signs of positive selection in our analysis, were highly conserved and indeed divergent between backgrounds and foregrounds (Fig. S4).

### Structural properties and expressional profiles

**Figure 5:**
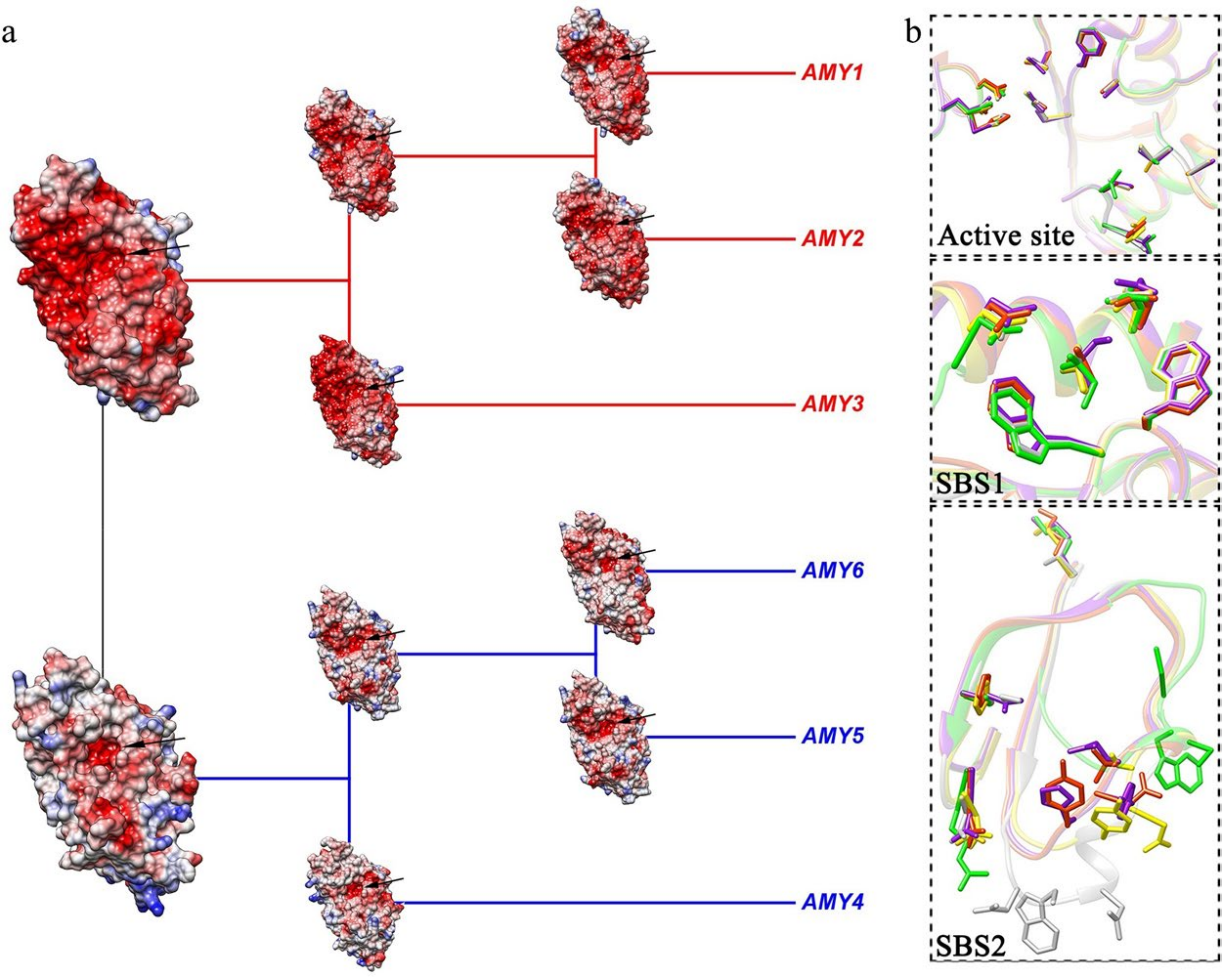
3D analyses of AMY protein structure. (**a**) Evolution of the divergent ancestral models across AMY1 to AMY6. Surfaces were colored with red, white and blue, separately indicating the negative (−10 kcal/mol·e, neutral (0) and positive (10 kcal/mol·e electrostatic potentials. (**b**) Structure comparison of substrate binding sites (the active site, SBS1 and SBS2). AMY1 was colored with gold, AMY2 with orange red, AMY3 with yellow, AMY4 with green, AMY5 with purple, and AMY6 with light gray. The active site of each ancestral node was inferred with black arrows. Residues involved in forming direct hydrogen bonds in substrate binding sites were summarized in Table S3.

The electrostatic potential reflects surface properties and molecular interactions that play critical roles in protein folding, conformational stability, enzyme catalysis and binding energies. Among the resulting models, apparent surface electrostatic changes were observed between the two main lineages, *AMY1* + *AMY2* + *AMY3* (negative surface potentials) and *AMY4* + *AMY5* + *AMY6* (positive to neutral potentials), in the central Domain A, in which the most essential active site of each ancestral node was consistently displayed as negative surface electrostatics (Fig. 5a). Further structural comparisons of the well-defined substrate binding sites indicated that the active site and SBS1 in Domain A shared highly similar structural folds across six *AMY* subfamilies, while the SBS2 displayed larger conformational variations (Fig. 5b). In each binding site, the amino acid residues involved in forming direct hydrogen bond contacts with starch-like substrates exhibited varied degree of conservations. For example, the catalytic residues (E205 and D291) in active site and the pair of consecutive tryptophans (W278 and W278) in SBS1 were conserved across subfamilies, whereas some other residues displayed degrees of substitutions or polymorphisms (Table S3).

We also examined expression patterns of *AMY* genes from rice, tomato, maize and *Arabidopsis* (Fig. S5). Generally, expression profiles of plant *AMY* genes varied across subfamilies in terms of developmental tissues and transcript abundance. In *Arabidopsis*, the single-copy *AMY3*, *AMY4* and *AMY5* genes expressed broadly in all the tissues sampled. Similar constitutive expression scenarios were observed in cases of *AMY1* to *AMY5* from maize, *AMY3* (Solyc03g095710) to *AMY6* from tomato, and *AMY4* and *AMY5* from rice. By contrast, the other *AMY3* copy (Solyc04g078930) in tomato was hardly detected in particular tissues (i.e., young leaves, young flower buds and anthesis flower 0DPA), and the *AMY1*, *AMY2* and *AMY3* paralogs in rice also displayed strong tissue-specific patterns. Even within the *AMY3* subfamily in rice, each of these four copies exhibited distinct expression profiles.

## Discussion

Alpha-amylase genes comprise four subtypes in cereal grasses and three in dicot species. Several phylogenetic studies restricted to higher model plants have been done^30,34,37,40,54,58^, however, a detailed sequence-derived comparison of these subfamilies is lacked, and little is known about how they evolved crossing the entire green lineage. With the goal of bridging this gap, we have examined the distribution, expansion dynamics, and potential functional differentiations using 472 redundant protein sequences, sampling from 78 different species or strains covering green algae to higher angiosperms.

Generally, *AMY1* and *AMY2* are grass-specific subfamilies. The previous defined *AMY1s* in dicots, such as At4g25000^37^ and *StAmy1*^54^, together with grass *AMY3*^30,34,40,58^, actually belong to plant *AMY3* lineage. The same case is *AMY4*, which consists of the *AMY2s* in dicots^37,54^ and *AMY4s* in grass^35,39,40,58^. The *AMY5* subfamily refers to the well-studied *AMY3* (At1g69830) in *Arabidopsis*, and its orthologous in other green plants. In addition, we have identified a novel subfamily (*AMY6*), which presents a scattered distribution from green algae to angiosperms (Fig. 1).

The N-terminal presequences of α-amylases have been well-described previously, and could be used as good signatures for discriminating genes belonging to specific subfamilies. In most cases, α-amylases encoded by *AMY1*, *AMY2* and *AMY3* genes are typical secretory proteins found in plastids (chloroplasts in leaves and amyloplasts in starchy cells)^49,50,52^, including a length of 24 to 27 signal peptides. However, those *AMY3* genes from the basal land plants (Liverworts, Mosses and Ferns) are predicted to carry the chloroplast signals. This clear signal shift from basal land plants to angiosperms may reflect some evolutionary novelties, such as the occurrence of seeds in plant bodies. Generally, the AMY4 isoform do not possess any signal peptide^39^. Our predictions indicate that three categories of signal patterns (secretory signals, no signals and chloroplast transit peptides) are overwhelmingly dominated the N-terminal regions of α-amylase genes in higher plants (Fig. S2). The most remarkable are AMY5 and AMY6, both of which own the N-terminal stretches of greater than 460 amino acids, and are predicted to carry the chloroplast signals. In green algae, the N-terminal signal divisions are less clear, because the mitochondrial targeting peptide and chloroplast transit peptide are always predicted to co-exist within each subfamily, whereas the secretory signals are absent. This discrepancy between green algae and land plants may be reflected by their divergence over a billion years ago, such as differences in photosynthetic and other critical metabolic pathways^59^. The transit peptide is necessary for plastidial targeting and translocation initiation^50^, thus, α-amylases with different signals suggest their different subcellular locations for starch digestion.

Both *AMY1* and *AMY2* genes are embedded in and belong to the grass *AMY3* lineage (Fig. 2b). This agrees well with the point that gene duplication leads to the formation of AmyA (*AMY1* and *AMY2*) and AmyB (*AMY3*) classes in the monocot lineage^34^. Colinearity identifications indicate that the *AMY1* loci originated from the ancestral cereal chromosome A2^57^, and *AMY2* from A6 (Fig. S3). Note that the chromosome A2 was the product of A4/A6 breakages and fusions^55,56^. So *AMY1* and *AMY2* genes should share a common single-copy locus from A6. To some extent, their evolutionary rates and expansion dynamics are similar. Meanwhile, the expansion of grass *AMY3* genes is also evident. Phylogenetic and syntenic relationships indicate that tandem duplications and whole genome duplications are keys to enlarge it from the archetype *AMY3* cluster of *3b* + *3c*.

With the radiation of grasses, numerous specific changes have happened, such as the acquisition of novel features (i.e., the timing of embryo development, and structures of flowers and fruits)^60^. Pineapple (*Ananas comosus*) belongs to the Bromeliaceae family that diverged from the grass family (Poaceae) 100 million years ago (MYA), making it a close relative for cereal genome evolution^61^. The absence of *AMY1* and *AMY2* genes in it, and even in more ancient basal sister groups such as banana (*Musa acuminata*), Date palm (*P. dactylifera*) and African oil palm (*E. guineensis*), provides useful clues for timing the birth of *AMY1* and *AMY2* genes. Together with the broad distribution of *AMY3* genes from green algae to higher plants (Fig. 1), we suggest that the common ancestor of *AMY1* and *AMY2* should be derived from the *AMY3* genes.

Previous studies well characterized the none-signal bearing *AMY4* and chloroplast-targeting *AMY5* genes. They unanimously agree that each of them represented a distinct *AMY* subfamily in angiosperms^54^. Differently, Mascher *et al*.^40^ put them together as a single subfamily (*AMY4* as *AMY4-1*, and *AMY5* as *AMY4-2*). In the present work, we provide more detailed identifications across the entire green lineage. In the closely-related Chlamydomonadales (*Chlamydomonas reinhardtii*, *Dunaliella salina*, *Volvox carteri*) and Sphaeropleales (*Monoraphidium neglectum*)^62^, *AMY5* genes are absent. Interestingly, their corresponding *AMY4* gene members (*Green algae_AMY4* in Fig. 2c) situate at the common ancestral position of *AMY4* and *AMY5* subfamilies. The poorly resolved or conflicting relationships of this ancestral branch may represent the ancestral status of *AMY4* and *AMY5* genes. The shared common ancestor is additionally explained by their equivalent chromosomal locations in genomes of four Brassicaceae species (*A*. *thaliana*, *A*. *lyrata*, *B*. *oleracea* and *B*. *rapa*) and *Prunus persica*. Thus, *AMY4* and *AMY5* should have resulted from an ancient gene duplication event following the radiation of Chlamydomonadales species, and the divergent direction is *AMY5* from *AMY4*. The newly described *AMY6* is distinct from *AMY5* based on their N-terminal features (Fig. S2). And from its distribution, we see that species with *AMY6* genes must have corresponding *AMY5* gene members, except those four basal land and vascular plants (Common liverwort, Moss, Bog moss and Spikemoss) (Fig. 1).

**Figure 6:**
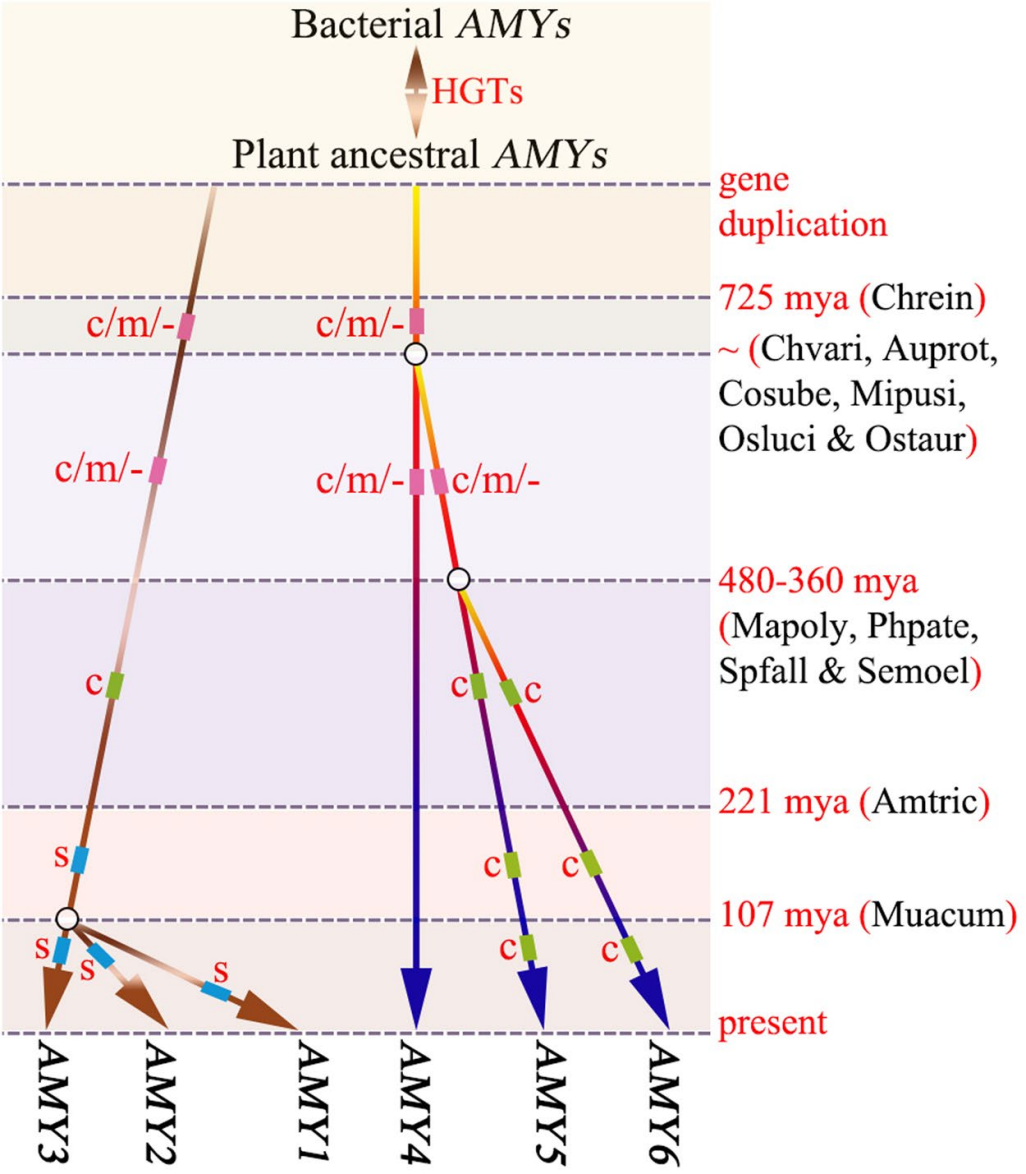
A proposal for the monophyletic origin of plant *AMY* genes. The ancestral archetype *AMY* gene in plants was initially duplicated into two primary lineages (*AMY3* and *AMY4*), which possibly exist predating green algal diversification. Circles represent three critical nodes for the birth of *AMY5*, *AMY6*, *AMY1* and *AMY2*, respectively. Rectangles along each line indicate different categories of signals, such as ‘c’ for chloroplast transit, ‘m’ for mitochondrial targeting, and ‘s’ for secretory pathways. Ages (mya: million years ago) of critical nodes for the evolution of green plants were based on previous publications^90–92^. The sister species were listed in parentheses.

Inter-kingdom comparisons provide important evidences for evolution of α-amylase genes in plants. Similar to the case of animals^4,9^, our inter-kingdom analysis indicates frequent horizontal gene transfers between plant and bacteria *AMY* genes (Fig. 4a), and the *AMY4* subfamily is more similar to bacterial *AMYs* than *AMY3* (Fig. 4b). That is to say, the plant *AMY* genes share a common ancestor, and *AMY4* genes have retained more sequence features of the ancestral *AMY*s in plants. In species of *C. reinhardtii*, *Aquilegia coerulea*, *Theobroma cacao* and *Cucumis sativus*, the equivalent chromosomal locations of *AMY3* and *AMY4* genes suggest gene duplications predating the diversification of green plants. Among those, we propose a diagram for the monophyletic origin of plant *AMYs* (Fig. 6).

In general, genes with constitutive expressions are more conserved than those exhibiting tissue-specific patterns^63,64^. The broad expressions of *AMY4* and *AMY5* genes among representative taxa (Fig. S5) suggest that they are functionally-essential in plants. Based on signal peptide predictions, we guess that these two subfamilies possibly target different subcellular localizations for initiating starch degradation, which is partly reflected by previous studies^37,54,65^. The novel *AMY6* with the closest phylogenetic relationship with *AMY5* also shares the similar constitutive expression scenario, however, it is scattered distributed, and absent or loss in particular green lineages (Fig. 1). These observations truly reflect the taxa-specific characteristic of *AMY6* genes. Cereal α-amylases, especially those encoded by *AMY1*, *AMY2* and *AMY3* genes, are typical secretory proteins during seed germination or grain development. In wheat and barley, *AMY1* and *AMY2* showed clear expression divergence in terms of transcript abundance, specific tissues or developmental stages^29^, and the wheat *AMY3* (Traest_5A1 in Fig. 3a) shared a similar pattern with *AMY2* in developing grains^30,31^. Unexpectedly, the expression of archetype *AMY3* genes (Traest_5A2, Traest_5B3, Traest_5D2 and Hovulg_5Hr1g068350) were hardly detected in all the tissues sampled (Data collection from https://wheat-urgi.versailles.inra.fr/ and http://webblast.ipk-gatersleben.de/barley_ibsc/, respectively), implying their nonfunctionalization during evolution. In rice, the expression divergence is also evident between *AMY1*and *AMY2*, and the four duplicated *AMY3* paralogs have more diversified expression profiles than single- or two-copy status in other representative organisms (Fig. S5).

The combination of sequence comparison, selection simulation and expression analysis leads to a definition of functional divergent between subfamilies. Hypothetically, the presence of multiple divergent isoforms could enable plant organisms to exploit categories of starch-like polysaccharides in a broad environmental conditions. Structural comparison further indicate that the active site and SBS1 are well-conserved (Fig. 5), which is not restricted to the cereal isoforms AMY1 and AMY2^42,66^, but among these six divergent enzymes (AMY1-AMY6). Generally, the active site is responsible for binding and catalyzing substrates^41,42,66^, whereas the secondary biding site (SBS1) and the AMY2-specific binding site (SBS2) are just critical for binding different starch-like polysaccharides^67,68^. This conservation reflect the ability in starch digestion across *AMY* subfamilies. However, the electrostatic changes between *AMY1* + *AMY2* + *AMY3* and *AMY4* + *AMY5* + *AMY6* (Fig. 5), together with the substituted residues involved in forming direct hydrogen bands (Table S3), may suggest the evolutionary diversification of enzyme specificity, such as substrate preference and product specificity. Furthermore, in the N-terminal presequences of *AMY5* genes, there also exist another kind of noncatalytic carbohydrate-binding module (CBM45), which was reported to be associated with plastidial starch metabolism^47^.

## Methods

### Sequence data

As previously reported by some authors^34,39,58^ and demonstrated by Yu *et al*.^69^, *AMY* genes were categorized into *AMY1* to *AMY4* among cereal crops and *AtAMY1* to *AtAMY3* in *A*. *thaliana*. For systemically identification of *AMYs* in green plants, we selected seven well-characterized *AMY* genes as queries (four GenBank accessions: CAX51372, CAX51374, AAA34259 and CAX51375 in barley and wheat, and three Araport11 entries: AT4G25000, AT1G76130 and AT1G69830 in *A*. *thaliana*) to perform blastp searches against databases of Phytozome v12.1 (https://phytozome.jgi.doe.gov/pz/portal.html), KEGG (http://www.genome.jp/), IPK Barley (http://webblast.ipk-gatersleben.de/barley_ibsc/), and blastn searches against Wheat URGI (https://wheat-urgi.versailles.inra.fr/) with default setting details, separately including 63, 13, 1 and 1 green plant species (Table S1). The deduced amino acid sequences were subjected to multiple sequence alignment by Clustalx^70^ and Bioedit^71^. Sequences not belonging to GH13_6, and/or with apparent sequence erosions (poor coverage and qualities) were discarded. For copy number determination, the primary peptide was chosen if alternative splicing peptides per copy were available. Since the N- and C-terminal end sections showed poor alignment across different *AMY* subfamilies, they were cutoff before phylogenetic reconstruction. And when several loci per species and several copies per locus were available, a single protein/copy sequence was kept if these loci/copies were shown to cluster in the same lineage. Subsequently, we selected 19 protein sequences representing all the analyzed *AMY* subfamilies across major plant lineages, to detect protein domain architecture using HMMER^72^. All the searches run with default parameters. Signal peptides were also predicted using the TargetP 1.1 server^73,74^.

### Phylogenetic reconstructions

In the first step, we analyzed the phylogenetic relationships between different subfamilies. We conducted Neighbor-Joining (NJ), Maximum Likelihood (ML) and Bayesian inference (BI) analyses using the single-copy orthologous dataset, including 309 protein sequences of functional *AMY* genes and 5 bacteria outgroups retrieved from NCBI (https://www.ncbi.nlm.nih.gov/) according to Da Lage *et al*.^9^. The NJ tree was analyzed using Jones-Taylor-Thornton (JTT) model with Gamma Distributed (G) rates across sites in MEGA7^75^. Branch confidence levels were estimated by using 1000 bootstrap replications. The ML trees were conducted using the best-fitting amino-acid model LG + G with the lowest Bayesian Information Criterion (BIC) score in MEGA7. Supports values were estimated from 500 non-parametric bootstrap iterations. The BI analyses were performed using MrBayes 3.2^76^. The preset Poisson substitution model was invoked by ‘Whelan and Goldman’ (WAG) through estimating the fixed rate models implemented in MrBayes 3.2. The option for rates was set to invgamma, and all the other parameters of the likelihood model were default values. Four simultaneous Markov chains (3 heated and 1 cold) were run starting from a random tree for 3 million generations requested and trees were sampled per 1,000 generations. The standard deviation of split frequencies fell below 0.05. Using a relative burn-in of 25% for diagnostics, the consensus tree was based on the remaining 1,500 trees. The confidence level of the tree topologies was estimated according to Bayesian posterior probabilities (BPPs). Gaps or missing data were treated as complete deletion.

Horizontal gene transfer (HGT) is the exchange of genetic material between organisms that are not in a parent-offspring relationship^77^. HGT was observed between animals and bacteria *AMY* genes^9^. For understanding the origin of green plant *AMY* genes, we compared protein sequences from eukaryotes (animals, plants, fungi and red algae) with those from bacteria. Firstly, we texted the key word ‘alpha-amylase’ against the protein fields of NCBI, and filtered with Swissprot entries. This strategy produced 87 animal and 79 fungal *AMYs*, and based on the global alignment, we selected 13 representative sequences from animals and 10 from fungi. Three *AMYs* in *A*. *thaliana* were selected as representatives in green plants. In red algae, we selected the single periplasmic sequence (Gasu_48600) in *G*. *sulphuraria* from KEGG as the representative. Then, we used all these representative sequences to blast against the KEGG prokaryote genomes. The top 10 hits with the annotations of ‘Alpha-amylase, EC: 3.2.1.1’ were kept. Finally, a dataset of 48 sequences from different living kingdoms (Table S2) were produced and used for NJ phylogenetic analysis with MEGA7. Supports values were estimated by using 1000 bootstrap replications.

### Synteny detection

Based on the well-established model of cereal genome evolution^55,56^, chromosome-scale pseudomolecules carrying *AMY* targets were downloaded from available resources for local database construction. Reciprocal blast was carried out to confirm the orthologous relationships^78^. By manual chromosomal walking, genic markers flanking *AMY* targets were used as queries to blast against the local database using the basic tool NCBI-BLAST-2.4.0+^79^. Genomic segments covering these markers were selected for gene order detection.

### Maximum likelihood analyses of positive selection

To measure variation in functional constraints and to test whether positive selection was involved among evolutionary *AMY* gene lineages, we estimated the omega values (ω = dN/dS, the ratio of the rate of nonsynonymous substitution per nonsynonymous site [dN] to the rate of synonymous substitution per synonymous site [dS]) through using a maximum-likelihood based CODEML program implemented in the PAML, version 4.9^80,81^. The branch models allowed the ω ratio to vary among lineages, and were specified for detecting adaptive selection acting on particular branches (variable ‘model’ and NSsites = 0)^82^. The site analysis allowed ω to vary among codon sites but kept it constant over the tree topologies (model = 0 and variable ‘NSsites’)^83^. The alternative Model A, also the recommended branch-site test of positive selection, assume that sites in predefined foreground branches are allowed to evolve under positive selection (ω_2_ > 1), whereas the background branches evolve neutrally (ω_1_ = 1) or under purifying selection (0 < ω_0_ < 1)^84,85^. Model A was tested against the nearly neutral site model M1a (Chi-square test, degree of freedom [df] = 2) using the likelihood ratio test (LRT)^82^. The Bayes Empirical Bayes (BEB) approach was used to calculate posterior probabilities and to select sites from the site class with ω > 1^86^. The codon dataset consisted of 41 sequences with 1137 characters. It was output from the PAL2NAL server (http://www.bork.embl.de/pal2nal/), and then used for model simulations. The phylogenetic subtree was presented in Fig. S1.

### 3D structure analysis and expression level comparison

To evaluate potential functional changes among different *AMY* subfamilies, we carried out 3D structure studies for each ancestral node back to the time point of gene divergence or duplication. A total of 10 ancestral amino acid sequences (AMY1 to AMY6, AMY12, AMY123, AMY56 and AMY456) were generated using parsimony state reconstruction in Mesquite 3.31 (http://mesquiteproject.org). We modeled protein crystal structures using the ancestral state sequences in the workplace of SWISS-MODEL^87^ based on their respective best matching templates from RCSB Protein Database Bank (PDB), such as template entries 1BG9^41^, 1RPK^66^, 2QPU^88^ and 3WN6^43^. The resulting models were then subjected to UCSF Chimera^89^ for electrostatic potential mapping (Coulombic surface coloring defaults: ε = 4r, thresholds ± 10 kcal/mol·e), domain comparison and visualization.

Four species (tomato, rice, *Arabidopsis* and maize) were selected for expressional analyses. Transcript profiles from various tissues in different developmental stages were retrieved from their corresponding databases: TFGD (http://ted.bti.cornell.edu/), Rice Genome Annotation Project (http://rice.plantbiology.msu.edu/), and *Arabidopsis* and maize eFP Browser (http://bar.utoronto.ca/efp/cgi-bin/efpWeb.cgi, http://bar.utoronto.ca/efp_maize/cgi-bin/efpWeb.cgi). The normalized expression values were log2 transformed. Heatmaps were generated by hierarchically clustering with the software R version 3.4.3 (https://www.R-project.org/).

## Supplementary information

supplementary information
