# Supplementary material for: New insights into the origin and evolution of α-amylase genes in green plants
Source: Sci Rep. 2019 Mar 20;9:4929. doi: 10.1038/s41598-019-41420-w (PMC6426938; doi:10.1038/s41598-019-41420-w)
Supplement: Supplementary file 1 — supplementary information [file 41598_2019_41420_MOESM1_ESM.pdf]

New insights into the origin and evolution of α-amylase genes in green plants

Liangliang Ju[1, 2], Zhifen Pan[1], Haili Zhang[1], Qiao Li[1], Junjun Liang[1], Guangbing Deng[1], Maoqun Yu[1], Hai Long[1]*

[1] Chengdu Institute of Biology, Chinese Academy of Sciences, Chengdu 610041, China

[2] University of Chinese Academy of Sciences, Beijing 100049, China

***Correspondence**: E-mail: hailong@cib.ac.cn (Tel: 86-028-82890325; Fax: 86-028-82890325)

Supplementary figure legends

Fig. S1 Analysis of selective pressures in the different branches of the phylogenetic tree. The one-ratio hypothesis that all the subfamilies are under the same selective pressures is not listed. The alternative two-ratio hypotheses that subfamilies *AMY1* to *AMY6* are accordingly as the foreground branches, which are predefined to be under selective pressures.

Fig. S2 Domain architecture of plant *AMY* subfamilies. Note that H_kinase_N refers to the signal transduction histidine kinase, which is found in bacteria but there is little published reference. Sequences used in it are consecutively CAX51374, CAX51372, Traest_5A1, Traest_4B1, Traest_5A2, AT4G25000, Spfall_0095s0050, Mapoly_0033s0036, Cosube_28437, CAX51375, AT1G76130, Spfall_0196s0001, Osluci_35756, Chrein_08g362450, Seital_5G295100, AT1G69830, Osluci_46693, Vivini_01032922001 and Spfall_0033s0066.

Fig. S3 Syntenic relationships of *AMY2* loci in grass. The *AMY2* copies are highlighted with red color.

Fig. S4 PAL2NAL output alignment of 41 amino acid sequences involved in selection detection. Residues under significant selection pressures in tests were colored with blue, and those that failed to detect but indeed divergent and positively selected were colored with black.

Fig. S5 Expression patterns of *AMY* genes in various tissues from different developmental stages of the four plant species. Expressional data of Solyc04g082090 in young leaves is missed. The color palette shades from light yellow with weak or no

expression to red with strong expression in RGB space with 100 unique colors.

Table S1 Summary information of plant taxa used in this work.

Species	Common name	Abbrivation	Phylogenetic lineage	Database
Chlamydomonas reinhardtii	Green algae	Chrein	Green algae	Phytozome
Dunaliella salina	Dunaliella	Dusali	Green algae	Phytozome
Volvox carteri	Volvox	Vocart	Green algae	Phytozome
Monoraphidium neglectum	NA	Monegl	Green algae	KEGG
Chlorella variabilis	NA	Chvari	Green algae	KEGG
Auxenochlorella protothecoides	NA	Auprot	Green algae	KEGG
Coccomyxa subellipsoidea	NA	Cosube	Green algae	Phytozome
Micromonas pusilla	NA	Mipusi	Green algae	Phytozome
Micromonas sp. RCC299	NA	Microm	Green algae	Phytozome
Ostreococcus lucimarinus	NA	Osluci	Green algae	Phytozome
Ostreococcus tauri	NA	Ostaur	Green algae	KEGG
Marchantia polymorpha	Common liverwort	Mapoly	Liverworts	Phytozome
Physcomitrella patens	Moss	Phpate	Mosses	Phytozome
Sphagnum fallax	Bog moss	Spfall	Mosses	Phytozome
Selaginella moellendorffii	Spikemoss	Semoel	Ferns	Phytozome
Amborella trichopoda	Amborella	Amtric	Basal angiosperms	Phytozome
Dendrobium catenatum	NA	Decate	Monocots	KEGG
Spirodela polyrhiza	Greater duckweed	Sppoly	Monocots	Phytozome
Zostera marina	Common eelgrass	Zomari	Monocots	Phytozome
Elaeis guineensis	African oil palm	Elguin	Monocots	KEGG
Phoenix dactylifera	Date palm	Phdact	Monocots	KEGG
Ananas comosus	Pineapple	Ancomo	Basal Poales	Phytozome
Musa acuminata	Banana	Muacum	Basal Poales	Phytozome
Brachypodium distachyon	Purple false brome	Brdist	Grass	Phytozome
Brachypodium stacei	NA	Brstac	Grass	Phytozome
Triticum aestivum	Bread wheat	Traest	Grass	IWGSC
Hordeum vulgare	Barley	Hovulg	Grass	IPK
Oryza sativa	Rice	Orsati	Grass	Phytozome
Oropetium thomaeum	NA	Orthom	Grass	Phytozome
Panicum hallii	Hall's panicgrass	Pahall	Grass - Panicoideae	Phytozome
Panicum virgatum	Switchgrass	Pavirg	Grass - Panicoideae	Phytozome
Setaria italica	Foxtail millet	Seital	Grass - Panicoideae	Phytozome
Setaria viridis	Green foxtail	Seviri	Grass - Panicoideae	Phytozome
Sorghum bicolor	Sorghum	Sobico	Grass - Panicoideae	Phytozome
Zea mays	Maize	Zemays	Grass - Panicoideae	Phytozome
Aquilegia coerulea	Colorado blue columbine	Aqcoer	Basal eudicots	Phytozome
Nelumbo nucifera	Sacred lotus	Nenuci	Core eudicots	KEGG
Amaranthus hypochondriacus	Amaranth	Amhypo	Core eudicots	Phytozome
Beta vulgaris	Sugar beet	Bevulg	Core eudicots	KEGG
Sesamum indicum	Sesame	Seindi	Core eudicots - Asterid	KEGG

Ipomoea nil	Japanese morning glory	Iponil	Core eudicots - Asterid	KEGG
Daucus carota	Carrot	Dacaro	Core eudicots - Asterid	Phytozome
Mimulus guttatus	Monkey flower	Migutt	Core eudicots - Asterid	Phytozome
Solanum lycopersicum	Tomato	Solyco	Core eudicots - Asterid	Phytozome
Solanum tuberosum	Potato	Sotube	Core eudicots - Asterid	Phytozome
Kalanchoe fedtschenkoi	diploid Kalanchoe	Kafedt	Core eudicots	Phytozome
Kalanchoe laxiflora	Milk widow's thrill	Kalaxi	Core eudicots	Phytozome
Eucalyptus grandis	Rose gum	Eugran	Core eudicots - Rosid	Phytozome
Vitis vinifera	Common grape vine	Vivini	Core eudicots - Rosid	Phytozome
Linum usitatissimum	Flax	Liusit	Core eudicots - Malpighiales	Phytozome
Manihot esculenta	Cassava	Maescu	Core eudicots - Malpighiales	Phytozome
Ricinus communis	Castor bean	Ricomm	Core eudicots - Malpighiales	Phytozome
Populus trichocarpa	Poplar	Potric	Core eudicots - Malpighiales	Phytozome
Salix purpurea	Purple osier willow	Sapurp	Core eudicots - Malpighiales	Phytozome
Citrus sinensis	Sweet orange	Cisine	Core eudicots - Citrus	Phytozome
Citrus clementina	Clementine	Ciclem	Core eudicots - Citrus	Phytozome
Carica papaya	Papaya	Capapa	Core eudicots - Malvales	Phytozome
Gossypium raimondii	Cotton	Goraim	Core eudicots - Malvales	Phytozome
Theobroma cacao	Cacao	Thcaca	Core eudicots - Malvales	Phytozome
Tarenaya hassleriana	Spider flower	Tahass	Core eudicots - Malvales	KEGG
Arabidopsis halleri	NA	Arhall	Core eudicots - Brassicaceae	Phytozome
Arabidopsis lyrata	Lyrate rockcress	Arlyra	Core eudicots - Brassicaceae	Phytozome
Arabidopsis thaliana	Thale cress	Arthal	Core eudicots - Brassicaceae	Phytozome
Boechera stricta	Drummond's rock cress	Bostri	Core eudicots - Brassicaceae	Phytozome
Brassica oleracea	Savoy cabbage	Broler	Core eudicots - Brassicaceae	Phytozome
Brassica rapa	Turnip mustard	Brrapa	Core eudicots - Brassicaceae	Phytozome
Capsella grandiflora	NA	Cagran	Core eudicots - Brassicaceae	Phytozome
Capsella rubella	Pink shepherd's purse	Carube	Core eudicots - Brassicaceae	Phytozome
Eutrema salsugineum	Salt cress	Eusals	Core eudicots - Brassicaceae	Phytozome
Cucumis sativus	Cucumber	Cusati	Core eudicots - Fabidae	Phytozome
Ziziphus jujuba	Chinese jujube	Zijuju	Core eudicots - Fabidae	KEGG
Fragaria vesca	Strawberry	Frvesc	Core eudicots - Fabidae	Phytozome
Malus domestica	Apple	Madome	Core eudicots - Fabidae	Phytozome
Prunus persica	Peach	Prpers	Core eudicots - Fabidae	Phytozome
Glycine max	Soybean	Glmax	Core eudicots - Fabidae	Phytozome
Phaseolus vulgaris	Common bean	Phvulg	Core eudicots - Fabidae	Phytozome
Medicago truncatula	Barrel medic	Metrun	Core eudicots - Fabidae	Phytozome
Trifolium pratense	Red clover	Trprat	Core eudicots - Fabidae	Phytozome

Table S2 ***AMY*** **genes used in the inter-kingdom comparisons.**

Category	Definition of Organisms	Abbriviation	Entry	Database
**Animal**	*Drosophila melanogaster* (Fruit fly)		P08144	UniProtKB
**Animal**	*Homo sapiens* (Human)		P19961	UniProtKB
Bacteria	*Alteromonas addita*	aaw	AVL56_17100	KEGG
Bacteria	*Pseudoalteromonas atlantica*	pat	Patl_2859	KEGG
Bacteria	*Colwellia* sp. PAMC 20917	coz	A3Q34_04185	KEGG
Bacteria	*Melittangium boletus* DSM 14713	mbd	MEBOL_007055	KEGG
Bacteria	*Stigmatella aurantiaca* DW4/3-1	sur	STAUR_7765	KEGG
Bacteria	*Cystobacter fuscus* DSM 52655	cfus	CYFUS_003156	KEGG
Bacteria	*Marinobacter salarius* R9SW1	msr	AU15_04180	KEGG
Bacteria	*Chloroflexus aurantiacus* J-10-fl	cau	Caur_3528	KEGG
Bacteria	*Herpetosiphon aurantiacus* DSM 785	hau	Haur_4065	KEGG
Bacteria	*Marinobacter* sp. LQ44	mlq	ASQ50_10800	KEGG
Bacteria	*Chloroflexus* sp. Y-400-fl	chl	Chy400_3804	KEGG
**Fungi**	*Thanatephorus cucumeris* (strain AG1-IB)		M5BQS7	UniProtKB
Bacteria	*Bacillus coagulans* DSM 1 = ATCC 7050	bcoa	BF29_2200	KEGG
Bacteria	*Bacillus coagulans* 36D1	bag	Bcoa_1344	KEGG
Bacteria	*Paenibacillus* sp. Y412MC10	gym	GYMC10_6058	KEGG
Bacteria	*Paenibacillus yonginensis* DCY84	pyg	AWM70_20460	KEGG
Bacteria	*Bacillus flexus* KLBMP 4941	bfx	BC359_01555	KEGG
Bacteria	*Exiguobacterium* sp. AT1b	eat	EAT1b_0567	KEGG
Bacteria	*Acetoanaerobium sticklandii* DSM 519	cst	CLOST_0052	KEGG
Bacteria	*Akkermansia muciniphila* ATCC BAA-835	amu	Amuc_1812	KEGG
Bacteria	*Sebaldella termitidis* ATCC 33386	str	Sterm_2808	KEGG
Bacteria	*Leptotrichia sp. oral taxon* 498 F0590	leq	BCB68_06425	KEGG
**Plant**	*Arabidopsis thaliana*	Arthal	AT1G76130	Phytozome
**Plant**	*Arabidopsis thaliana*	Arthal	AT1G69830	Phytozome
**Plant**	*Arabidopsis thaliana*	Arthal	AT4G25000	Phytozome
Bacteria	*Cystobacter fuscus* DSM 52655	cfus	CYFUS_001925	KEGG
Bacteria	*Stigmatella aurantiaca* DW4/3-1	sur	STAUR_2365	KEGG
Bacteria	*Melittangium boletus* DSM 14713	mbd	MEBOL_004825	KEGG
Bacteria	*Archangium gephyra* DSM 2261	age	AA314_02608	KEGG
Bacteria	*Spirochaeta thermophila* DSM 6578	stq	Spith_1658	KEGG
Bacteria	*Spirochaeta thermophila* DSM 6192	sta	STHERM_c15980	KEGG
Bacteria	*Cellvibrio japonicus* Ueda107	cja	CJA_3126	KEGG
Bacteria	*Saccharophagus degradans* 2-40	sde	Sde_0563	KEGG
Bacteria	*Archangium gephyra* DSM 2261	age	AA314_03843	KEGG
Bacteria	*Corallococcus coralloides* DSM 2259	ccx	COCOR_02752	KEGG
**Red algae**	*Galdieria sulphuraria*	gsl	Gasu_48600	KEGG
Bacteria	*Sorangium cellulosum* So ce 56	scl	sce5091	KEGG
Bacteria	*Chondromyces crocatus* Cm c5	ccro	CMC5_062270	KEGG
Bacteria	*Candidatus Solibacter usitatus* Ellin6076	sus	Acid_4507	KEGG

Bacteria	*Nitrospira japonica* NJ1	nja	NSJP_2858	KEGG
Bacteria	*Streptomyces lincolnensis* NRRL 2936	sls	SLINC_1229	KEGG
Bacteria	*Cyanothece* sp. PCC 8802	cyh	Cyan8802_2152	KEGG
Bacteria	*Leptolyngbya* sp. NIES-3755	len	LEP3755_14340	KEGG
Bacteria	*Planctomyces* sp. SH-PL14	pls	VT03_23095	KEGG
Bacteria	*Singulisphaera acidiphila* DSM 18658	saci	Sinac_7048	KEGG
Bacteria	*Pseudanabaena* sp. PCC 7367	pseu	Pse7367_0286	KEGG

Table S3 Residues involved in forming direct hydrogen bonds in substrate binding sites.

Substrate bingding	Template (1rpk)	Parsimony ancestral state reconstruction					
		AMY1	AMY2	AMY3	AMY4	AMY5	AMY6
Active site	H-45	Q-42	H-42		Q-40	E-41	E-40
	V-47	V-44			L-42	V-43	V-42
	C-95	T-92	C-92		V-89	C-90	C-89
	A-96	A-93			G-90	A-91	A-90
	A-146	A-143			G-132	A-135	A-134
	R-178	R-175			R-164	R-167	R-166
	F-181	F-178			F-167	F-170	F-169
	E-205	E-202			E-191	E-194	E-193
	H-290	H-286	H-287	H-284	H-272	H-276	H-274
	D-291	D-287	D-288	D-285	D-273	D-277	D-275
	Q-296	Q-292	Q-293	Q-290	Q-278	Q-282	Q-280
SBS1	Q-227	Q-224			Q-212	Q-215	Q-214
	V-230	V-227			I-215	I-218	V-217
	D-234	D-231			D-219	N-222	N-221
	W-278	W-274	W-275	W-272	W-260	W-264	W-262
	W-279	W-275	W-276	W-273	W-261	W-265	W-263
SBS2	K-375	K-367	K-368	K-365	K-355	K-356	K-355
	Y-380	Y-376	Y-377	Y-374	S-364	H-365	D-364
	D-381	D-377	D-378	D-375	W-365	Y-366	W-365
	V-382	V-378	V-379	V-376	C-366	E-367	N-366
	H-395	H-391	H-392	S-389	C-377	E-380	K-383
	D-398	D-394	D-395	D-392	R-380	D-383	D-386

Residue collection is based on previous comparative structural studies [1, 2]. Residue numbering is according to the template sequence of 1rpk.

References

1. Robert, X., et al., *The Structure of Barley α-Amylase Isozyme 1 Reveals a Novel Role of Domain C in Substrate Recognition and Binding.* Structure, 2003. **11**(8): p. 973-984.
2. Robert, X., et al., *Oligosaccharide binding to barley α-amylase 1.* Journal of Biological Chemistry, 2005. **280**(38): p. 32968-32978.

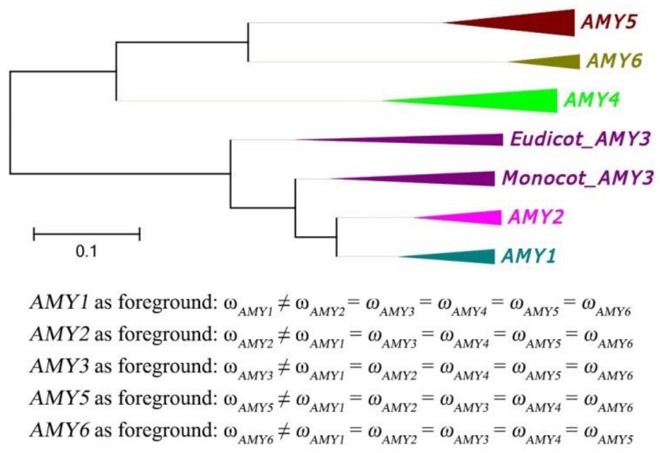

Fig. S1 Analysis of selective pressures in the different branches of the phylogenetic tree. The one-ratio hypothesis that all the subfamilies are under the same selective pressures is not listed. The alternative two-ratio hypotheses that subfamilies *AMY1* to *AMY6* are accordingly as the foreground branches, which are predefined to be under selective pressures.

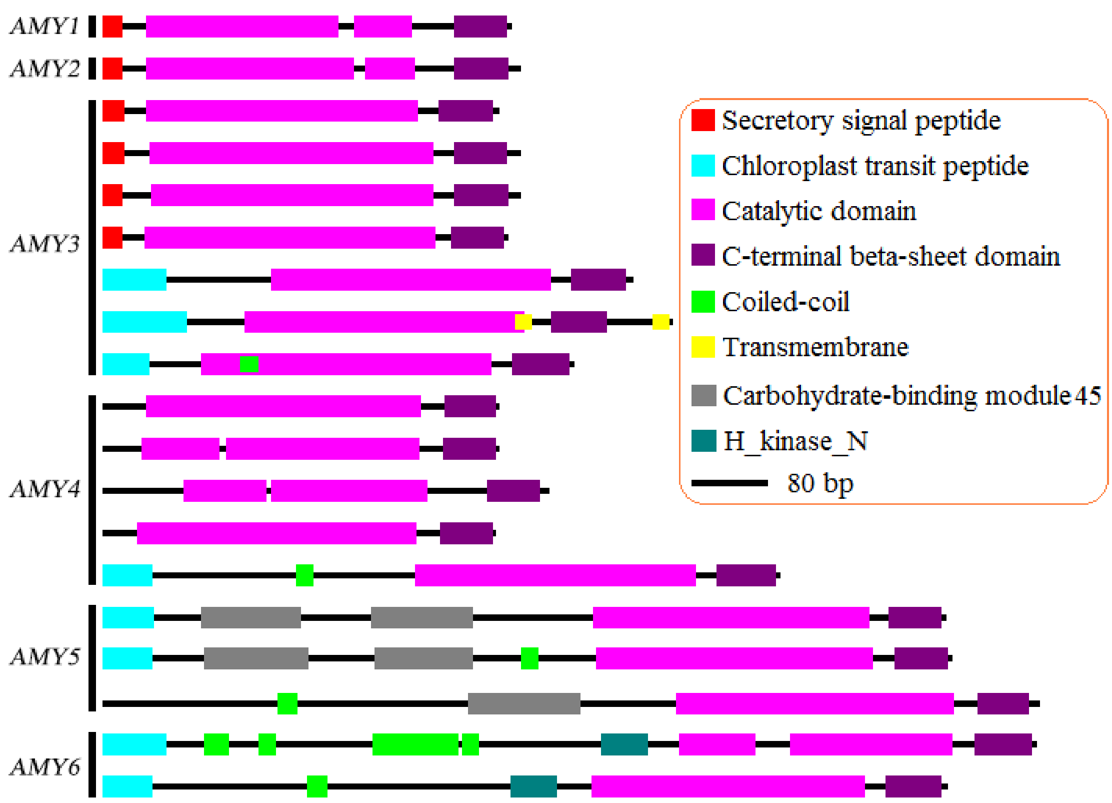

Fig. S2 Domain architecture of plant *AMY* subfamilies. Note that H_kinase_N refers to the signal transduction histidine kinase, which is found in bacteria but there is little published reference. Sequences used in it are consecutively CAX51374, CAX51372, Traest_5A1, Traest_4B1, Traest_5A2, AT4G25000, Spfall_0095s0050, Mapoly_0033s0036, Cosube_28437, CAX51375, AT1G76130, Spfall_0196s0001, Osluci_35756, Chrein_08g362450, Seital_5G295100, AT1G69830, Osluci_46693, Vivini_01032922001 and Spfall_0033s0066.

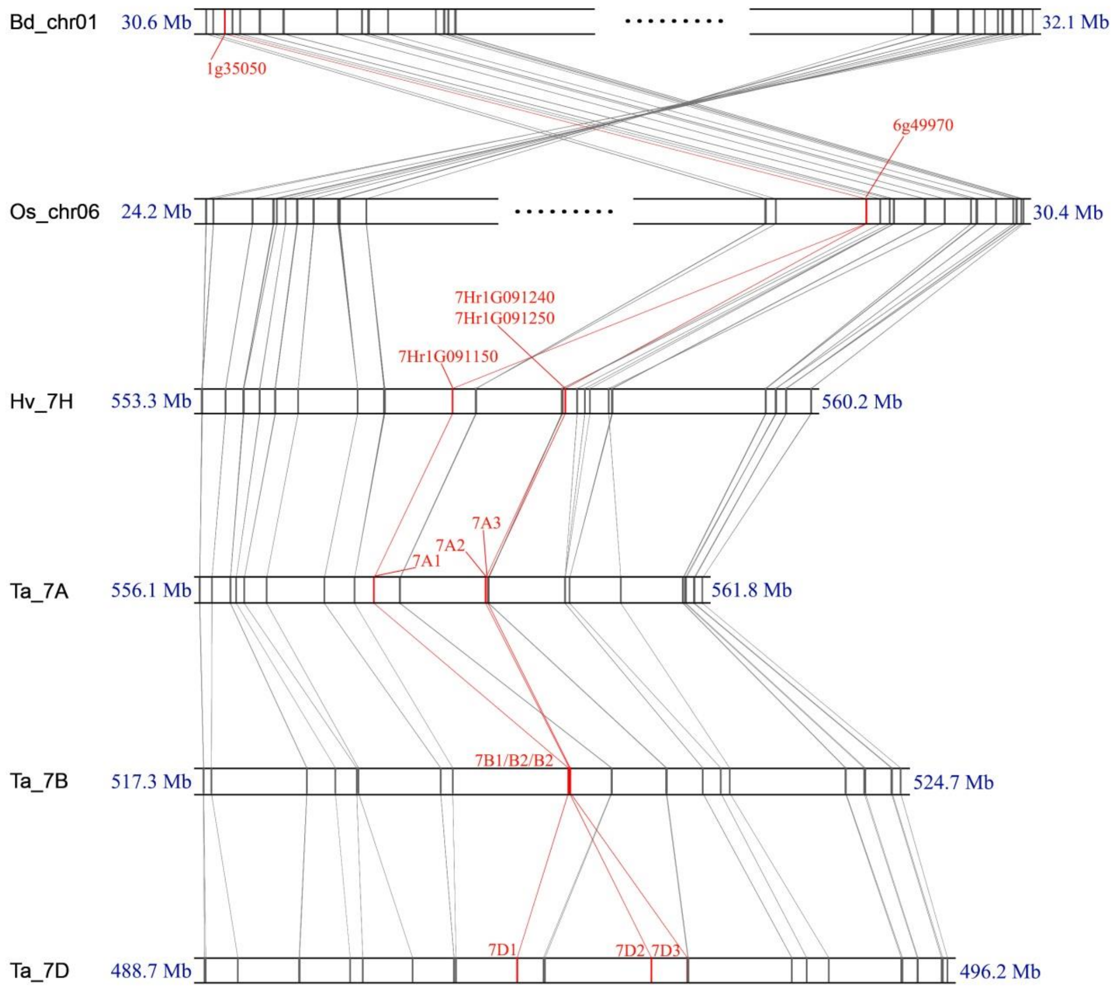

Fig. S3 Syntenic relationships of *AMY2* loci in grass. The *AMY2* copies are highlighted with red color.

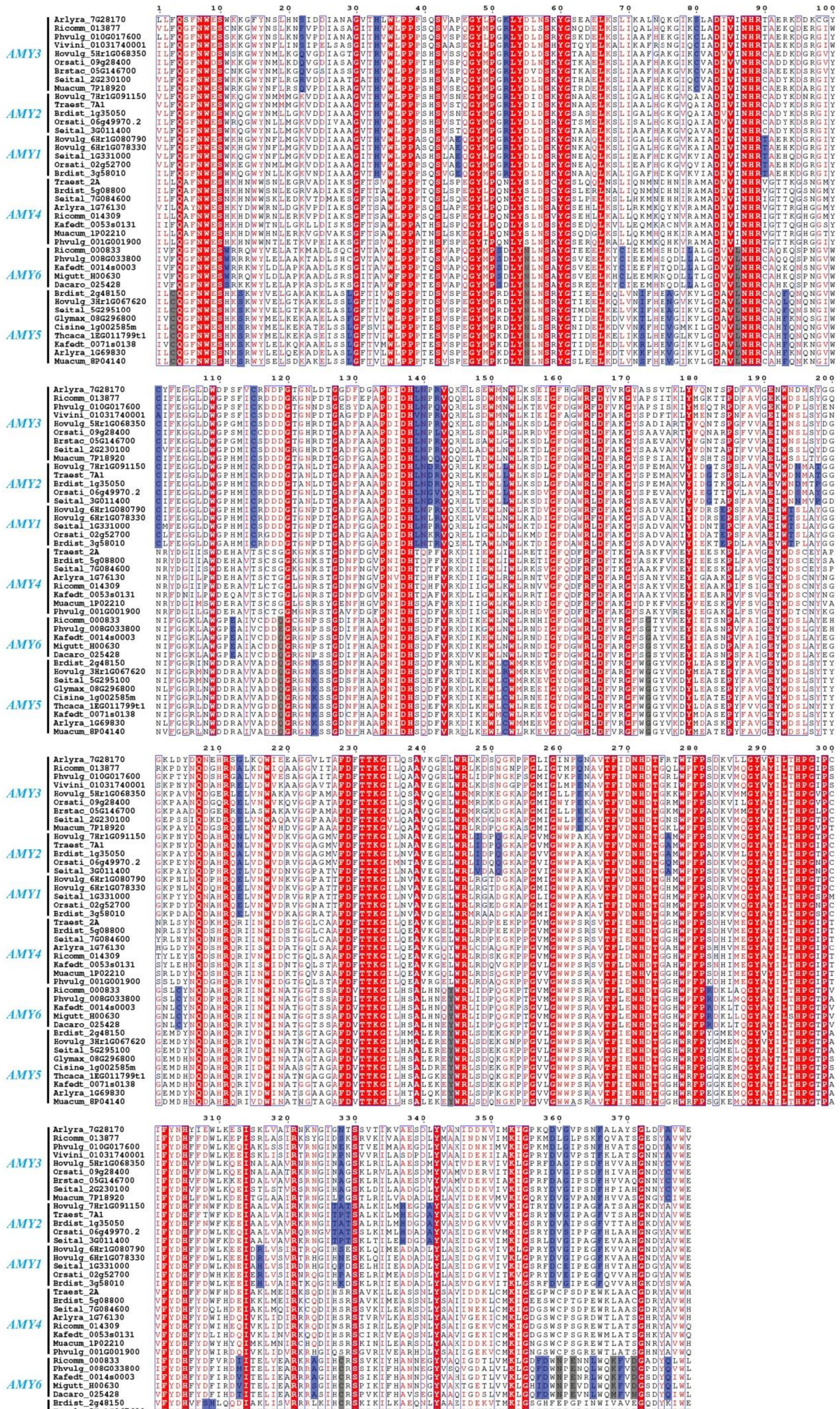
AMY3
AMY2
AMY1
AMY4
AMY6
AMY5

Fig. S4 PAL2NAL output alignment of 41 amino acid sequences involved in selection detection. Residues under significant selection pressures in tests were colored with blue, and those that failed to detect but indeed divergent and positively selected were colored with black.

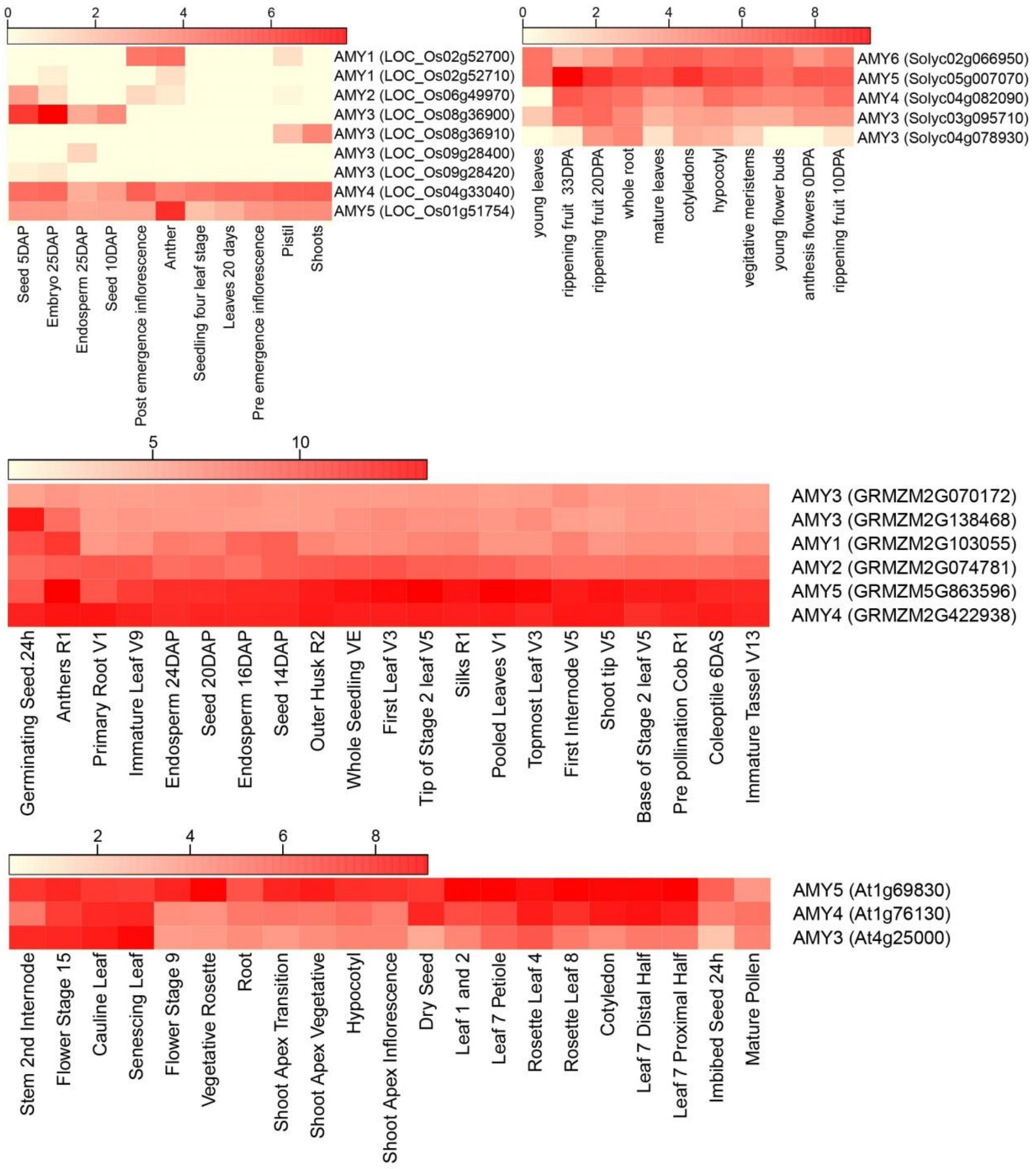

Fig. S5 Expression patterns of *AMY* genes in various tissues from different developmental stages of the four plant species. Expressional data of Solyc04g082090 in young leaves is missed. The color palette shades from light yellow with weak or no expression to red with strong expression in RGB space with 100 unique colors.
